# An 8-gene mRNA expression profile in circulating tumor cells predicts response to aromatase inhibitors in metastatic breast cancer patients

**DOI:** 10.1186/s12885-016-2155-y

**Published:** 2016-02-18

**Authors:** Esther A. Reijm, Anieta M. Sieuwerts, Marcel Smid, Joan Bolt-de Vries, Bianca Mostert, Wendy Onstenk, Dieter Peeters, Luc Y. Dirix, Caroline M. Seynaeve, Agnes Jager, Felix E. de Jongh, Paul Hamberg, Anne van Galen, Jaco Kraan, Maurice P. H. M. Jansen, Jan W. Gratama, John A. Foekens, John W. M. Martens, Els M. J. J. Berns, Stefan Sleijfer

**Affiliations:** Department of Medical Oncology and Cancer Genomics Netherlands, Erasmus MC – Cancer Institute, Erasmus University Medical Center, Room He 116, P.O. Box 2040, Rotterdam, 3000 CA The Netherlands; Translational Cancer Research Unit, Oncology Center GZA Hospitals Sint-Augustinus and Department of Oncology, University of Antwerp, Antwerp, Belgium; Department of Internal Medicine, Ikazia Hospital, Rotterdam, The Netherlands; Department of Internal Medicine, Sint Franciscus Gasthuis, Rotterdam, The Netherlands

**Keywords:** Breast cancer, Circulating tumor cells, Aromatase inhibitors, Predictive profile

## Abstract

**Background:**

Molecular characterization of circulating tumor cells (CTC) is promising for personalized medicine. We aimed to identify a CTC gene expression profile predicting outcome to first-line aromatase inhibitors in metastatic breast cancer (MBC) patients. *Methods:* CTCs were isolated from 78 MBC patients before treatment start. mRNA expression levels of 96 genes were measured by quantitative reverse transcriptase polymerase chain reaction. After applying predefined exclusion criteria based on lack of sufficient RNA quality and/or quantity, the data from 45 patients were used to construct a gene expression profile to predict poor responding patients, defined as disease progression or death <9 months, by a leave-one-out cross validation.

**Results:**

Of the 45 patients, 19 were clinically classified as poor responders. To identify them, the 75 % most variable genes were used to select genes differentially expressed between good and poor responders. An 8-gene CTC predictor was significantly associated with outcome (Hazard Ratio [HR] 4.40, 95 % Confidence Interval [CI]: 2.17–8.92, *P* < 0.001). This predictor identified poor responding patients with a sensitivity of 63 % and a positive predictive value of 75 %, while good responding patients were correctly predicted in 85 % of the cases. In multivariate Cox regression analysis, including CTC count at baseline, the 8-gene CTC predictor was the only factor independently associated with outcome (HR 4.59 [95 % CI: 2.11–9.56], *P* < 0.001). This 8-gene signature was not associated with outcome in a group of 71 MBC patients treated with systemic treatments other than AI.

**Conclusions:**

An 8-gene CTC predictor was identified which discriminates good and poor outcome to first-line aromatase inhibitors in MBC patients. Although results need to be validated, this study underscores the potential of molecular characterization of CTCs.

**Electronic supplementary material:**

The online version of this article (doi:10.1186/s12885-016-2155-y) contains supplementary material, which is available to authorized users.

## Background

Metastatic breast cancer (MBC) is a highly heterogeneous disease leading to an urgent need for a more personalized treatment approach. For those patients with estrogen receptor (ER)-expressing tumors, endocrine therapy is the mainstay of treatment. Although many patients greatly benefit from such endocrine therapies, approximately 30 % of the MBC patients never respond while virtually all initial responders eventually relapse and develop progressive disease. Numerous factors accounting for resistance to endocrine treatment have been revealed, including loss of ER expression [1–3], overexpression of the HER2 receptor [4], hyperactivation of the phosphatidylinositol 3-kinase (PI3K) pathway [5], and overexpression of Enhancer of Zeste Homolog 2 (EZH2) [6]. Determination of these factors in tumor tissue may therefore contribute to a more personalized treatment approach of individual patients.

Predictive factors contributing to treatment decision making are nowadays most commonly identified in the primary tumors. However, heterogeneity in molecular characteristics between primary tumor and metastases, including clinically relevant factors, is increasingly recognized. For example, differences in ER expression between primary tumor and metastases occur in approximately 20 % of the patients leading to treatment changes in a substantial number of patients [1, 7, 8]. Since this heterogeneity increases over time and under treatment pressure [7], repetitive analyses of the characteristics of metastatic tumor cells are likely to offer better guidance of treatment choices than characterization of the primary tumor. Unfortunately, metastatic tissue is often hard to obtain and only possible through invasive procedures.

Circulating tumor cells (CTCs) are tumor cells found in the peripheral blood and are thought to better represent the actual or clinically relevant metastatic tissue burden than the primary tumor does, in particular in those patients whose primary tumors have been removed several years prior to diagnosis of MBC. The CTC count has shown to be a powerful prognostic factor in MBC and a rise or decline in CTC count after the first cycle of systemic therapy is an early predictor of outcome [9–12]. Additionally, CTC characterization holds great promise and for that purpose, several techniques to molecularly characterize CTCs for drug target expression [13–15], mutations [16] and gene expression [17–19] have been developed. CTCs however occur in relatively low numbers in patients with MBC and, even after the epithelial cell adhesion molecule (EpCAM)-based enrichment of the CellSearch® system, they need to be identified and characterized amongst approximately a thousand of remaining leukocytes [20]. This greatly hinders the interpretation of results from techniques non-selective for tumor cells such as quantitative reverse transcriptase polymerase chain reaction (qRT-PCR) on whole lysates. Nevertheless, by focusing on genes that are not, or only at a much lower level, expressed by leukocytes, we have previously shown that the expression levels of 96 genes in CTCs can be quantified in MBC patients through qRT-PCR [18].

In this study, we aim to quantify this panel of 96 genes in CTCs of MBC patients with ER-expressing primary tumors prior to start of first-line therapy with an aromatase inhibitor (AI) in order to identify a CTC predictor discriminating between good and poor responders.

## Methods

### Ethics statement

This study has been approved by the medical ethics committee of the Erasmus MC Rotterdam, The Netherlands and local Institutional Review Boards (ethics boards of Oncology Center GZA Hospitals Sint-Augustinus, Antwerp, Belgium; Ikazia Hospital, Rotterdam, The Netherlands; Sint Franciscus Gasthuis, Rotterdam) (METC 2006–248 and METC 2009–405). All patients gave their written informed consent.

We adhered to the Reporting Recommendations for Tumor Marker Prognostic Studies wherever possible [21].

### Collection of blood samples and characteristics of recruited patient cohort

MBC patients had been included between October 2008 and August 2012 in 5 hospitals. From 78 MBC patients who were not previously treated for MBC and prior to start of first-line AI therapy (irrespective of type), 2 × 7.5 mL blood samples were prospectively drawn for CTC enumeration and isolation. Due to insufficient RNA quality and/or quantity and/or lack of expression of previously described CTC-specific genes [18] (for details see next), 33 (42 %) samples were excluded, providing 45 patients for further analysis (Additional file 1: Figure S1). Detailed clinicopathological information for these 45 patients is provided in Table 1.

**Table 1:** Patients and their clinico-pathological characteristics

Characteristic	No. of patients	%
All patients	*45*	*100 %*
Time between primary surgery and CTC sampling (DFI)		
≤ 5 years	*16*	*36 %*
> 5 years	*21*	*47 %*
Primary not removed	*8*	*18 %*
Age at CTC sampling		
≤ 50 years	*4*	*9 %*
> 50 years	*41*	*91 %*
Menopausal status		
Premenopausal	*2*	*4 %*
Postmenopausal	*43*	*96 %*
Histologic grade (Bloom-Richardson)		
I, well differentiated	*5*	*11 %*
II, moderately differentiated	*23*	*51 %*
III, poorly differentiated	*4*	*9 %*
Unknown	*13*	*29 %*
Pathological tumor size		
pT1, ≤2 cm	*20*	*44 %*
pT2-4, >2 cm	*22*	*49 %*
Unknown	*3*	*7 %*
Lymph nodes involved		
No	*14*	*31 %*
Yes	*27*	*60 %*
Unknown	*4*	*9 %*
ERa status^a^		
Negative	*1*	*2 %*
Positive	*44*	*98 %*
PgR status^a^		
Negative	*5*	*11 %*
Positive	*36*	*80 %*
Unknown	*4*	*9 %*
HER2/neu status^a^		
Negative	*37*	*82 %*
Positive	*3*	*7 %*
Unknown	*5*	*11 %*
Histological type		
Lobular	*13*	*29 %*
Ductal	*28*	*62 %*
Ductolobular	*3*	*7 %*
Ductal, signet-cell	*1*	*2 %*
Adjuvant chemotherapy		
No	*31*	*69 %*
Yes	*14*	*31 %*
Adjuvant hormonal therapy		
No	*24*	*53 %*
Yes	*21*	*47 %*
Any adjuvant therapy		
No	*22*	*49 %*
Yes	*23*	*51 %*
Site of metastasis		
Visceral	*5*	*11 %*
Non-visceral	*26*	*58 %*
Both	*14*	*31 %*
1st line treatment		
Anastrozol	*15*	*33 %*
Letrozol	*16*	*36 %*
Exemestane	*14*	*31 %*
Median progression-free survival (PFS in days; range)^b^	358 (14–1255)	
Median baseline CTC count (range in 7.5 mL blood)	8 (0–32,492)	

^a^As retrieved from pathology reports

^b^Also includes censoring data from patients censored at last follow-up date

In order to be able to decipher whether obtained results from this AI-treated patient cohort are of prognostic or predictive nature, we used an independent patient cohort composed of 71 MBC patients that received other types of first-line therapy. Of these, 21 patients were treated with chemotherapy, 40 patients with chemotherapy combined with targeted therapy, and 10 patients with tamoxifen therapy. This patient cohort had been extracted from MBC patients described in our recently published study in which the same techniques for CTC enrichments and gene expression determination were applied [22].

### Enumeration of CTCs

In order to isolate CTCs for CTC enumeration, 7.5 mL blood was drawn in CellSave tubes (Veridex™ LCC, Raritan, NJ, USA) and processed on the CellTracks AutoPrep System by using the CellSearch Epithelial Cell Kit (both Veridex LCC). CTC enumeration was performed on the CellTracks Analyzer (Veridex LCC) according to the manufacturer’s instructions and as described previously [23–25].

### mRNA isolation from CTCs, qRT-PCR and quantification of gene transcripts

Together with the blood samples for CTC enumeration, another 7.5 mL blood of the same patients was drawn in EDTA tubes. These samples were enriched for CTCs on the CellTracks AutoPrep System using the CellSearch Profile Kit (Veridex LCC). Isolated cells were lysed by adding 250 μL of Qiagen AllPrep DNA/RNA Micro Kit Lysis Buffer (RLT+ lysis buffer) (Qiagen BV, Venlo, The Netherlands) and immediately stored at −80 °C until RNA isolation was performed with the AllPrep DNA/RNA Micro Kit (Qiagen) according to the manufacturer’s instructions and as previously described [18].

The generation of cDNA from isolated RNA from CTCs and subsequent pre-amplification and TaqMan-based PCR analysis were performed as described before [20]. The 96 measured mRNA transcripts have previously been selected and validated based on their clinical relevance and potential CTC-specificity [18, 20].

### Reference genes, data normalization, and quality control

The procedure of data normalization and quality control was performed as previously described [18, 20]. In short, relative expression levels were quantified by using the delta Ct method, which is the difference between the average Ct of the reference genes *HMBS*, *HPRT1*, and *GUSB* and the Ct of the target genes. Samples that were able to generate a signal within the chosen cut-off set at 26 Ct of the average of the reference genes were considered of sufficient quality and quantity to be included in the study and quantified for the levels of the remaining 93 target genes. By the use of this threshold, 5 of our initial 78 CTC samples (6 %) were excluded from further analysis.

Finally, samples were checked for sufficient expression levels of a 12-gene mRNA cluster that has previously been determined as epithelial-specific and associated with the presence of CTCs [18]. Due to lack of sufficient expression of these genes and our aim to generate a CTC-specific predictor, another 28 CTC samples (36 %) were excluded from further analysis.

### Statistical analysis

Statistical analyses were done with the STATA statistical package, release 12.0 (STATA Corp., College Station, TX). Primary endpoint was progression-free survival (PFS), defined as the time elapsed between start of first-line treatment with AI and clinical and/or radiological progression or death, whichever came first. Patients who were alive and had not progressed were censored at the last follow-up date, which was at least 9 months after start of 1st line therapy. Those patients with progression or death <9 months were considered as poor responders. This 9-month cut-off was chosen based on the median PFS for first-line therapy in MBC patients as reported in the literature [26, 27]. In all 45 eligible patients, a leave-one-out-cross validation (LOOCV) was conducted using the Support Vector Machines (SVM) method within Biometric Research Branc ArrayTools (http://linus.nci.nih.gov/BRB-ArrayTools.html) after selecting the top 75 % most variable genes from the 93 genes described above. With this LOOCV method, a gene signature was generated that consisted out of the most differentially expressed genes that were identified in the individual predictions and best predicted the left-out sample. A panel of 8 genes was identified that performed best in predicting the poor responding patients. The SVM method proved superior compared to the other prediction algorithms; based on 100 permutations, SVM was the only method with a significant *P*-value of 0.01. Cluster 3.0 and TreeView (http://bonsai.hgc.jp/~mdehoon/software/cluster/clustersetup.exe and http://jtreeview.sourceforge.net/ [28]) were used to cluster the samples according to the gene expression values of these 8 genes and to visualize the results. Survival curves were generated using the Kaplan-Meier method and a logrank test was used to test for differences. All statistical tests were 2-sided with *P* < 0.05 considered statistically significant.

## Results

### Patient characteristics

Characteristics of the 45 patients who were eligible for our CTC-specific analyses to explore differentially expressed genes between good and poor responders are listed in Table 1. One patient was described to have an ER-negative primary tumor but received hormonal treatment in both adjuvant and first-line setting due to PR-positivity. Median baseline CTC count in the 45 patient cohort was 8 (range 0 – 32,492 CTCs/7.5 mL blood). The extremely high CTC count of 32,492 was assessed in a patient who did not respond to treatment and died within one month after treatment initiation due to progression of disease. The 9-month cutoff as based on literature data on the median PFS in first-line MBC patients [26, 27] was well-chosen considering the median PFS of 11.8 months (range 0 – 41.3 months) in our 45 patient cohort.

### 8-gene CTC profile predicts for outcome to treatment

Of the 45 patients, 19 patients were classified as poor responders due to progression of disease or death <9 months whereas the remaining 26 patients were considered good responders. A LOOCV was performed in this cohort yielding an 8-gene predictor in which each gene had a univariate *P*-value of <0.1 (Table 2). Application of this 8-gene CTC profile resulted in 16 patients with an unfavorable profile and were thus predicted to be poor responders. Twelve of them truly showed resistance to therapy <9 months (disease progression or death) and four did not, resulting in a sensitivity of 63 % and a positive predictive value (PPV) of 75 % (Table 3). Applying the profile, 29 patients had a favorable profile and were thus predicted not to show progressive disease <9 months. Of these, 22 indeed did not fail treatment <9 months rendering a specificity of 85 % and a negative predictive value (NPV) of 76 %.

**Table 2:** Significantly differentially expressed genes between 45 good and poor responders

Gene	*P*-value	t-value
TWIST1	0,001	−2,879
KRT81	0,018	−2,453
PTRF	0,029	−2,024
EEF1A2	0,031	−1,895
PTPRK	0,049	−1,793
EGFR	0,065	−1,701
CXCL14	0,080	2,229
ERBB3	0,096	2,26

A negative t-value corresponds to higher expression in poor responding patients; a positive t-value to higher expression in good responding patients

**Table 3:** Test performance

	8-gene CTC profile		
PFS <9 months	Favorable	Unfavorable	Total
No	22	4	26
Yes	7	12	19
Total	29	16	45
Pearson’s *X* ^2^ statistic	10,93		
*P*	<0.001		

**Fig. 1:**
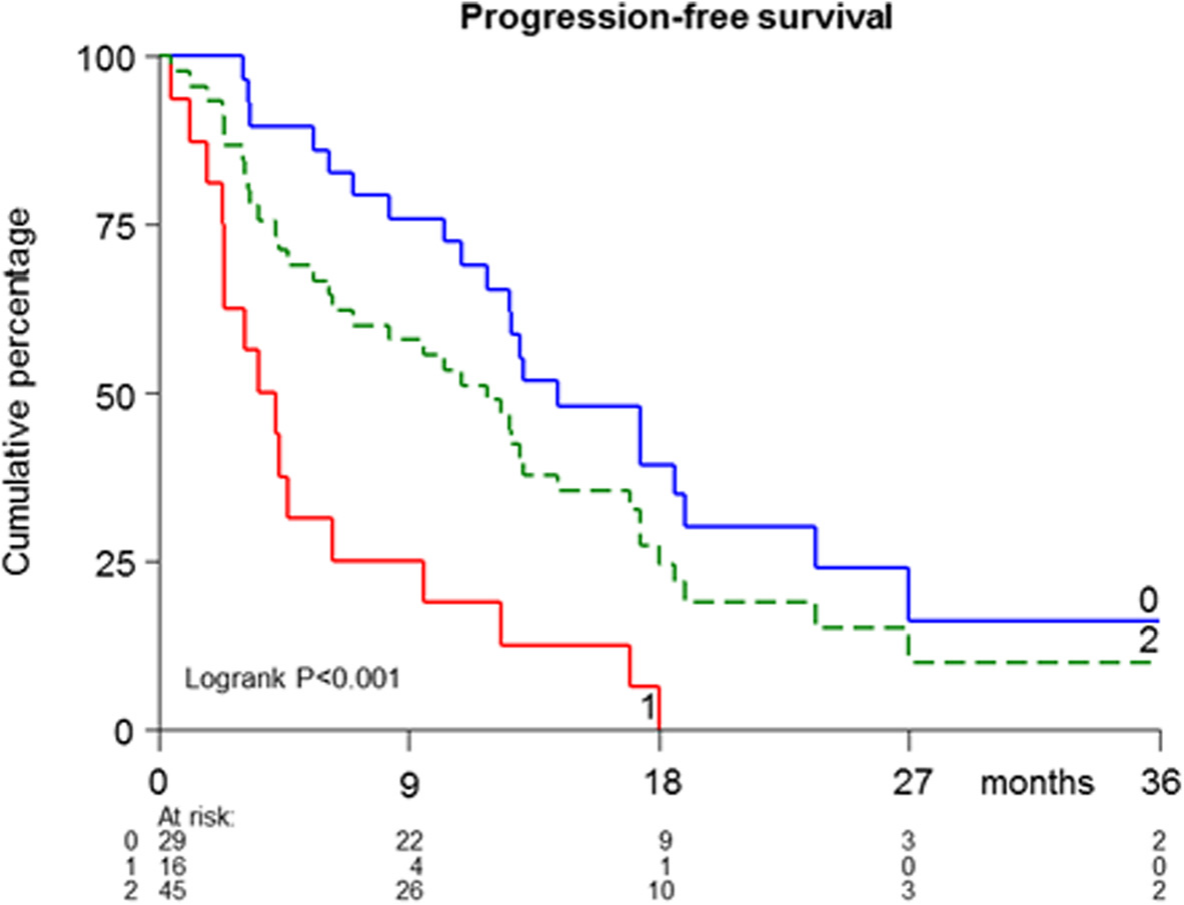
Kaplan-Meier curve for patients as defined by the 8-gene CTC predictor. *Blue (0): favorable profile; red (1): unfavorable profile; green (2): total cohort (N = 45)*

The Kaplan-Meier curves for PFS of the predicted good and poor responding patients according to the 8-gene CTC predictor are shown in Fig. 1 and were statistically different (Logrank *P* < 0.001).

In univariate analysis, the 8-gene CTC predictor was significantly associated with PFS (HR 4.40 [95 % CI: 2.17–8.92], *P* < 0.001). When including the traditional predictive factors, disease-free interval (DFI), which was defined as the time between primary surgery and CTC sampling, the dominant site of relapse, and the CTC count at baseline in a multivariate analysis, only the 8-gene CTC-profile was an independent predictor of PFS (HR 4.59 [95 % CI: 2.16–9.75], *P* < 0.001) (Table 4). The CTC count at baseline was not associated with PFS in this 45 patient cohort, but showed to be significant in the total cohort of 78 patients (HR 2.47 [95 % CI: 1.43-4.27], *P* = 0.001) (Additional file 2: Figure S2).

**Table 4:** Predictive value of the 8-gene CTC profile in uni- and multivariate analysis

Factor of base model	Univariate analysis			Multivariate analysis		
	PFS			PFS		
	HR	95 % CI	*P*	HR	95 % CI	*P*
<5 vs. ≥5 CTCs at baseline	1,11	0.57–2.15	0,753	1,31	0.65–2.62	0.455
Disease-free interval^a^	1,09	0.74–1.61	0,653	0,95	0.62–1.43	0.790
Dominant site of relapse^b^	1,34	0.69–2.58	0,384	1,11	0.56–2.18	0.768
8-gene CTC profile	4,40	2.17–8.92	<0.001	4,59	2.16–9.75	<0.001

^a^Defined as the time between primary surgery and CTC sampling and analyzed in 3 groups: ≤5 years (*N* = 12), >5 years (*N* = 21) and metastatic disease upon diagnosis (*N* = 12)

^b^Divided into non-visceral vs. visceral metastases

### Hierarchical clustering to identify clusters of patients according to the 8-gene CTC predictor

**Fig. 2:**
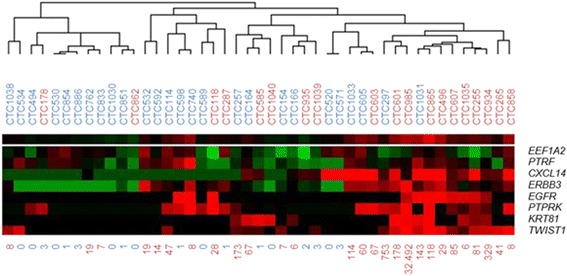
Unsupervised hierarchical cluster analysis comparing the 8-gene CTC predictor in 45 MBC patients treated with first-line AI therapy. Each horizontal row represents a gene, and each vertical column corresponds to a sample. Red color indicates a mRNA expression level above the median level, black color indicates a median expression level, and green color indicates an expression level below the median level of the assay as measured in all 45 samples. The number of CTCs as established by the CellSearch Epithelial Kit is depicted below the figure. *Blue: good responder; red: poor responder. CTC count: blue: <5 CTCs; red: ≥5 CTCs*

Two-dimensional average linkage hierarchical cluster analysis [28] was performed to compare the difference in gene expression of the 8 identified genes in our 45 patients. This analysis resulted in a clustering of 2 major and 5 minor groups of patients in which cluster 1 mainly contained the good responders (10 out of 12), whereas cluster 2 consisted of both good and poor responders (Fig. 2). In this cluster, however, a subcluster existed that, with 10 out of 12, predominantly contained poor responders with higher expression of most of the identified 8 genes.

### Testing the 8-gene CTC profile in an independent differently treated patient cohort

Having identified the 8-gene CTC profile in AI-treated patients, it was assessed whether this signature was prognostic or predictive by investigating the association between this profile and outcome in an independent patient cohort composed of 71 MBC patients that received other first-line therapies than AI. Of these, 21 patients were treated with chemotherapy, 40 with chemotherapy combined with a type of targeted therapy such as trastuzumab, and 10 with tamoxifen therapy. Of this group, 35 patients had a PFS of less than 9 months and were therefore classified as having a poor outcome. Application of the 8-gene CTC profile resulted in 33 patients with a favorable CTC profile. The CTC profile however, could not properly discriminate the patients with a good versus those with a poor outcome (*P* = 0.899; Table 5).

**Table 5:** Test performance of the 8-gene CTC predictor in 71 patients not treated with AI therapy

	8-gene CTC profile		
PFS <9 months	Favorable	Unfavorable	Total
No	16	19	35
Yes	17	19	36
Total	33	38	71
Pearson’s *X* ^2^ statistic	0,016		
*P*	0.899		

## Discussion

Characterization of CTCs holds great promise to predict response to treatment and to gain more insight into mechanisms underlying resistance to systemic anti-tumor agents. Although whole transcriptome analysis would be most preferable, isolation of CTCs by the CellSearch technique does not result in pure fractions of CTCs but only in fractions enriched for CTCs in which an overload of leukocytes is still present. This makes interpretation of whole transcriptome analysis impossible since only techniques yielding pure CTC fractions would allow such analyses. We have previously shown to be able to measure mRNA expression levels of multiple epithelial genes in CTCs enriched by CellSearch [18]. By using these selected genes and applying the same technique, the current study demonstrates the ability of using CTC characterization as a predictor for response to endocrine therapy. To our best knowledge, this is the first study that has generated an unique CTC-based gene expression panel that is able to distinguish good and poor responders to first-line AI therapy. From a clinical point of view, it is probably more relevant to identify the poor rather than the good responding patients, since these patients might benefit more from another treatment. Our identified 8-gene CTC profile however performed better in predicting the good responders, since the specificity of the predictor outperforms its sensitivity (85 % vs. 63 %; Table 3). Nevertheless, this could still impact clinical decision making since good responding patients could undergo less intensive follow-up strategies and fewer laboratory procedures which is not only less demanding for patients but can also reduce health care costs.

In order to explore whether this signature associated with outcome in AI-treated patients is prognostic or predictive, we tested the profile in CTCs of a group of 71 patients who were treated with types of systemic treatments other than AI including chemotherapy (*N* = 21), chemotherapy combined with a type of targeted therapy (*N* = 40), or tamoxifen therapy (*N* = 10). In contrast to the AI-treated patients, the 8-gene CTC profile could not discriminate patients with a good versus those with a poor outcome in this group of patients (*P* = 0.899; Table 5). Although this is not a true validation of the test, it strongly supports that the identified profile is predictive for outcome to AI therapy and not for outcome to other agents. It needs to be underscored that the identified CTC profile has been obtained in a small number of patients for which an LOOCV procedure to reveal such a profile is commonly applied. It important to realize that such an approach bears the risk of overfitting the data as a consequence of which validation in an independent patient cohort is needed before implementation in clinical practice.

The development of a CTC-specific predictor required exclusion of patients who lacked sufficient expression of epithelial-specific genes. These are mainly patients with no or few counted CTCs and are therefore more likely to have a longer PFS which might have biased our patient set [9]. Although most characteristics do not show differences between in- and excluded patients (Additional file 3: Table S1), the median PFS in the 33 excluded patients was 548 (40–1694) days which significantly differs from the median PFS of 358 (14–1255) days in the 45 included patients (Logrank *P* < 0.001). This exclusion criterion highly affected the number of patients available for further analysis. The low number of remaining patients might be the reason for the insignificant association between the CTC count at baseline (divided in <5 vs. ≥5 CTCs) and PFS. In the total cohort of 78 patients, CTC count was significantly related to PFS (Additional file 2: Figure S2). Since cohorts with few patients cannot be divided into independent discovery and validation sets, resampling the original data through cross-validation is statistically the best method [29].

Amongst the 8 genes that we found to be associated with outcome to AI therapy through LOOCV, was the epithelial marker *KRT81*. Many cytokeratins are highly expressed in both normal and tumor epithelium in which the pattern of expression can be used to identify the tissue of origin [30]. Not much is known about this specific cytokeratin and why high expression would lead to a worse outcome. Mutations in *KRT81* have been described in monilethrix, a condition in which patients develop diffuse hypothrichosis [31].

*CXCL14* and *ERBB3* were the only genes that were more abundantly expressed in the good responding patients. This is discordant to what is currently known in primary tumor tissue with respect to both genes. The published literature, however, only considers gene expression in primary tumors which cannot easily be extrapolated to CTCs. *CXCL14* is a chemokine that has been shown to be upregulated in tumor myoepithelial cells and enhances the proliferation, migration, and invasion of epithelial cells after binding to their receptors [32]. Expression of *ERBB3* has, similar to *EGFR* in our CTC predictor, previously been associated with endocrine therapy resistance when highly expressed in primary tumor tissue [33, 34]. The predictor also contained high expression of *PTRF* and *EEF1A2* to be associated with poor outcome. This is in contrast with previously published literature in which *PTRF* has been shown to interact with *pS2/TTF1* [35] which on its turn needs ER as key transcriptional factor in order to be expressed [36] and is associated with a better clinical outcome in breast cancer [37–39]. *EEF1A2* is an eukaryotic elongation factor of which its expression downregulates through interaction with protein p16 (INK4a) leading to inhibition of cancer cell growth [40]. It is mainly known as a potential oncogene in ovarian cancer in which its expression enhances cell growth in vitro [41]. Overexpression of *EEF1A2* has also been seen in breast tumors [42] and it is one of the genes in the 76-gene signature as identified in the ER-positive subset of 115 primary breast tumors that represent a strong prognostic factor for patients at high risk for developing metastases [43, 44]. With respect to the other genes of the predictor, *PTPRK* belongs to the group of protein-tyrosine phosphatases (PTPs) that control tyrosine phosphorylation. PTPs regulate the signaling of growth-factor receptors and can, when deregulated, be associated with tumorigenesis [45]. Deregulation of PTPs can result in both their up- and downregulation, which can explain the discordance between our established association between high expression of *PTPRK* and poor outcome to AI therapy, while decreased expression of *PTPRK* has previously been related to poor prognosis in MBC suggesting a more tumor suppressive role [46]. *TWIST1*, at last, is a transcription factor that is one of the most widely known factors to be involved in the process of epithelial-to-mesenchymal-transition (EMT). Its overexpression has been associated with endocrine therapy resistance due to downregulation of ER promoter activity [47]. Moreover, through direct repression of E-cadherin cells and activation of mesenchymal markers, *TWIST1* plays an essential role in tumor metastasis [48]. The appearance of *TWIST1* in our 8-gene CTC predictor is remarkable since our applied CTC isolation method relies on an EpCAM-based enrichment step and tumor cells undergoing EMT might become EpCAM-negative [49]. The dependency on EpCAM-expression by CTCs renders the CellSearch method therefore not the best method to capture all CTCs, but it is still the only FDA-cleared method which will enable its implementation and obtained results in clinical studies. In addition, whether EpCAM loss always accompanies EMT is still under debate [50].

Although ER is amongst the 93 target genes that were measured, its mRNA expression in this study was not associated with outcome to AI therapy. Several techniques have been explored to determine ER expression in CTC, but so far, none of these studies could show an association with outcome (reviewed in [19]). Recently, Babayan et al. have demonstrated the possibility of measuring ER protein expression in single CTCs through immunofluorescence. This study revealed that CTCs of individual MBC patients with ER-positive primary tumors are frequently a heterogeneous population consisting of both ER-positive and ER-negative CTCs [51]. Similar to primary tumor tissue, the percentage of ER-positive CTCs may be the best parameter associated with outcome rather than ER mRNA expression of the total CTC fraction as was measured in our study.

## Conclusion

In conclusion, we have here defined an 8-gene expression predictor established in CTCs that is associated with outcome to first-line AI therapy in MBC patients. Importantly, before the results of the current study can be implemented, an independent patient cohort is warranted to validate the results found here. Nevertheless, this study underscores the enormous potential that molecular characterization of CTCs has.

## Additional files

Additional file 1: Figure S1. Flowchart depicting the numbers of patients included and excluded from the study. (TIF 18.6 kb) Additional file 2: Figure S2. Kaplan-Meier curve as defined by CTC count for the total cohort of 78 patients. *Blue (0): <5 CTCs at baseline; red (1): ≥5 CTCs at baseline.* (XLSX 15 kb) Additional file 3: Table S1. Cohort of in- (*N* = 45) and excluded (*N* = 33) patients and their clinico-pathological characteristics. (TIF 23 kb)

## References

[1] Thompson AM, Jordan LB, Quinlan P, Anderson E, Skene A, Dewar JA (2010). Prospective comparison of switches in biomarker status between primary and recurrent breast cancer: the Breast Recurrence In Tissues Study (BRITS). Breast Cancer Res.

[2] Gong Y, Han EY, Guo M, Pusztai L, Sneige N (2011). Stability of estrogen receptor status in breast carcinoma: a comparison between primary and metastatic tumors with regard to disease course and intervening systemic therapy. Cancer.

[3] Amir E, Miller N, Geddie W, Freedman O, Kassam F, Simmons C (2012). Prospective study evaluating the impact of tissue confirmation of metastatic disease in patients with breast cancer. J Clin Oncol.

[4] Pancholi S, Lykkesfeldt AE, Hilmi C, Banerjee S, Leary A, Drury S (2008). ERBB2 influences the subcellular localization of the estrogen receptor in tamoxifen-resistant MCF-7 cells leading to the activation of AKT and RPS6KA2. Endocr Relat Cancer.

[5] Miller TW, Balko JM, Arteaga CL (2011). Phosphatidylinositol 3-kinase and antiestrogen resistance in breast cancer. J Clin Oncol.

[6] Reijm EA, Jansen MP, Ruigrok-Ritstier K, van Staveren IL, Look MP, van Gelder ME (2011). Decreased expression of EZH2 is associated with upregulation of ER and favorable outcome to tamoxifen in advanced breast cancer. Breast Cancer Res Treat.

[7] Campbell PJ, Yachida S, Mudie LJ, Stephens PJ, Pleasance ED, Stebbings LA (2010). The patterns and dynamics of genomic instability in metastatic pancreatic cancer. Nature.

[8] Xiao C, Gong Y, Han EY, Gonzalez-Angulo AM, Sneige N (2011). Stability of HER2-positive status in breast carcinoma: a comparison between primary and paired metastatic tumors with regard to the possible impact of intervening trastuzumab treatment. Ann Oncol.

[9] Cristofanilli M, Budd GT, Ellis MJ, Stopeck A, Matera J, Miller MC (2004). Circulating tumor cells, disease progression, and survival in metastatic breast cancer. N Engl J Med.

[10] Cristofanilli M, Hayes DF, Budd GT, Ellis MJ, Stopeck A, Reuben JM (2005). Circulating tumor cells: a novel prognostic factor for newly diagnosed metastatic breast cancer. J Clin Oncol.

[11] Hayes DF, Cristofanilli M, Budd GT, Ellis MJ, Stopeck A, Miller MC (2006). Circulating tumor cells at each follow-up time point during therapy of metastatic breast cancer patients predict progression-free and overall survival. Clin Cancer Res.

[12] Bidard FC, Peeters DJ, Fehm T, Nole F, Gisbert-Criado R, Mavroudis D (2014). Clinical validity of circulating tumour cells in patients with metastatic breast cancer: a pooled analysis of individual patient data. Lancet Oncol.

[13] Attard G, Swennenhuis JF, Olmos D, Reid AH, Vickers E, A'Hern R (2009). Characterization of ERG, AR and PTEN gene status in circulating tumor cells from patients with castration-resistant prostate cancer. Cancer Res.

[14] Fehm T, Muller V, Aktas B, Janni W, Schneeweiss A, Stickeler E (2010). HER2 status of circulating tumor cells in patients with metastatic breast cancer: a prospective, multicenter trial. Breast Cancer Res Treat.

[15] Riethdorf S, Muller V, Zhang L, Rau T, Loibl S, Komor M (2010). Detection and HER2 expression of circulating tumor cells: prospective monitoring in breast cancer patients treated in the neoadjuvant GeparQuattro trial. Clin Cancer Res.

[16] Gasch C, Bauernhofer T, Pichler M, Langer-Freitag S, Reeh M, Seifert AM (2013). Heterogeneity of epidermal growth factor receptor status and mutations of KRAS/PIK3CA in circulating tumor cells of patients with colorectal cancer. Clin Chem.

[17] Smirnov DA, Zweitzig DR, Foulk BW, Miller MC, Doyle GV, Pienta KJ (2005). Global gene expression profiling of circulating tumor cells. Cancer Res.

[18] Sieuwerts AM, Mostert B, Bolt-de Vries J, Peeters D, de Jongh FE, Stouthard JM (2011). mRNA and microRNA expression profiles in circulating tumor cells and primary tumors of metastatic breast cancer patients. Clin Cancer Res.

[19] Onstenk W, Gratama JW, Foekens JA, Sleijfer S (2013). Towards a personalized breast cancer treatment approach guided by circulating tumor cell (CTC) characteristics. Cancer Treat Rev.

[20] Sieuwerts AM, Kraan J, Bolt-de Vries J, van der Spoel P, Mostert B, Martens JW (2009). Molecular characterization of circulating tumor cells in large quantities of contaminating leukocytes by a multiplex real-time PCR. Breast Cancer Res Treat.

[21] McShane LM, Altman DG, Sauerbrei W, Taube SE, Gion M, Clark GM (2005). Reporting recommendations for tumor marker prognostic studies (REMARK). J Natl Cancer Inst.

[22] Mostert B, Sieuwerts AM, Kraan J, Bolt-de Vries J, van der Spoel P, van Galen A (2015). Gene expression profiles in circulating tumor cells to predict prognosis in metastatic breast cancer patients. Ann Oncol.

[23] Sieuwerts AM, Kraan J, Bolt J, van der Spoel P, Elstrodt F, Schutte M (2009). Anti-epithelial cell adhesion molecule antibodies and the detection of circulating normal-like breast tumor cells. J Natl Cancer Inst.

[24] Mostert B, Kraan J, Bolt-de Vries J, van der Spoel P, Sieuwerts AM, Schutte M (2011). Detection of circulating tumor cells in breast cancer may improve through enrichment with anti-CD146. Breast Cancer Res Treat.

[25] Kraan J, Sleijfer S, Strijbos MH, Ignatiadis M, Peeters D, Pierga JY (2011). External quality assurance of circulating tumor cell enumeration using the Cell Search((R)) system: a feasibility study. Cytometry B Clin Cytom.

[26] Gennari A, Conte P, Rosso R, Orlandini C, Bruzzi P (2005). Survival of metastatic breast carcinoma patients over a 20-year period: a retrospective analysis based on individual patient data from six consecutive studies. Cancer.

[27] Kiely BE, Soon YY, Tattersall MH, Stockler MR (2011). How long have I got? Estimating typical, best-case, and worst-case scenarios for patients starting first-line chemotherapy for metastatic breast cancer: a systematic review of recent randomized trials. J Clin Oncol.

[28] Eisen MB, Spellman PT, Brown PO, Botstein D (1998). Cluster analysis and display of genome-wide expression patterns. Proc Natl Acad Sci U S A.

[29] Molinaro AM, Simon R, Pfeiffer RM (2005). Prediction error estimation: a comparison of resampling methods. Bioinformatics.

[30] Moll R, Franke WW, Schiller DL, Geiger B, Krepler R (1982). The catalog of human cytokeratins: patterns of expression in normal epithelia, tumors and cultured cells. Cell.

[31] Ferrando J, Galve J, Torres-Puente M, Santillan S, Nogues S, Grimalt R (2012). Monilethrix: A New Family with the Novel Mutation in KRT81 Gene. Int J Trichol.

[32] Allinen M, Beroukhim R, Cai L, Brennan C, Lahti-Domenici J, Huang H (2004). Molecular characterization of the tumor microenvironment in breast cancer. Cancer Cell.

[33] Nicholson RI, Hutcheson IR, Harper ME, Knowlden JM, Barrow D, McClelland RA (2001). Modulation of epidermal growth factor receptor in endocrine-resistant, oestrogen receptor-positive breast cancer. Endocr Relat Cancer.

[34] Morrison MM, Hutchinson K, Williams MM, Stanford JC, Balko JM, Young C (2013). ErbB3 downregulation enhances luminal breast tumor response to antiestrogens. J Clin Invest.

[35] Jansa P, Mason SW, Hoffmann-Rohrer U, Grummt I (1998). Cloning and functional characterization of PTRF, a novel protein which induces dissociation of paused ternary transcription complexes. EMBO J.

[36] Metivier R, Penot G, Hubner MR, Reid G, Brand H, Kos M (2003). Estrogen receptor-alpha directs ordered, cyclical, and combinatorial recruitment of cofactors on a natural target promoter. Cell.

[37] Foekens JA, Rio MC, Seguin P, van Putten WL, Fauque J, Nap M (1990). Prediction of relapse and survival in breast cancer patients by pS2 protein status. Cancer Res.

[38] Foekens JA, van Putten WL, Portengen H, de Koning HY, Thirion B, Alexieva-Figusch J (1993). Prognostic value of PS2 and cathepsin D in 710 human primary breast tumors: multivariate analysis. J Clin Oncol.

[39] Foekens JA, Portengen H, Look MP, van Putten WL, Thirion B, Bontenbal M (1994). Relationship of PS2 with response to tamoxifen therapy in patients with recurrent breast cancer. Br J Cancer.

[40] Lee MH, Choi BY, Cho YY, Lee SY, Huang Z, Kundu JK (2013). Tumor suppressor p16(INK4a) inhibits cancer cell growth by downregulating eEF1A2 through a direct interaction. J Cell Sci.

[41] Pinke DE, Kalloger SE, Francetic T, Huntsman DG, Lee JM (2008). The prognostic significance of elongation factor eEF1A2 in ovarian cancer. Gynecol Oncol.

[42] Tomlinson VA, Newbery HJ, Wray NR, Jackson J, Larionov A, Miller WR (2005). Translation elongation factor eEF1A2 is a potential oncoprotein that is overexpressed in two-thirds of breast tumours. BMC Cancer.

[43] Wang Y, Klijn JG, Zhang Y, Sieuwerts AM, Look MP, Yang F (2005). Gene-expression profiles to predict distant metastasis of lymph-node-negative primary breast cancer. Lancet.

[44] Zhang Y, Sieuwerts AM, McGreevy M, Casey G, Cufer T, Paradiso A (2009). The 76-gene signature defines high-risk patients that benefit from adjuvant tamoxifen therapy. Breast Cancer Res Treat.

[45] Alonso A, Sasin J, Bottini N, Friedberg I, Friedberg I, Osterman A (2004). Protein tyrosine phosphatases in the human genome. Cell.

[46] Sun PH, Ye L, Mason MD, Jiang WG (2013). Protein tyrosine phosphatase kappa (PTPRK) is a negative regulator of adhesion and invasion of breast cancer cells, and associates with poor prognosis of breast cancer. J Cancer Res Clin Oncol.

[47] Vesuna F, Lisok A, Kimble B, Domek J, Kato Y, van der Groep P (2012). Twist contributes to hormone resistance in breast cancer by downregulating estrogen receptor-alpha. Oncogene.

[48] Yang J, Mani SA, Donaher JL, Ramaswamy S, Itzykson RA, Come C (2004). Twist, a master regulator of morphogenesis, plays an essential role in tumor metastasis. Cell.

[49] Iwatsuki M, Mimori K, Yokobori T, Ishi H, Beppu T, Nakamori S (2010). Epithelial-mesenchymal transition in cancer development and its clinical significance. Cancer Sci.

[50] van der Gun BT, Melchers LJ, Ruiters MH, de Leij LF, McLaughlin PM, Rots MG (2010). EpCAM in carcinogenesis: the good, the bad or the ugly. Carcinogenesis.

[51] Babayan A, Hannemann J, Spotter J, Muller V, Pantel K, Joosse SA (2013). Heterogeneity of estrogen receptor expression in circulating tumor cells from metastatic breast cancer patients. PLoS One.

