# Supplementary figures and images for: An 8-gene mRNA expression profile in circulating tumor cells predicts response to aromatase inhibitors in metastatic breast cancer patients
Source: BMC Cancer. 2016 Feb 18;16:123. doi: 10.1186/s12885-016-2155-y (PMC4759736; doi:10.1186/s12885-016-2155-y)

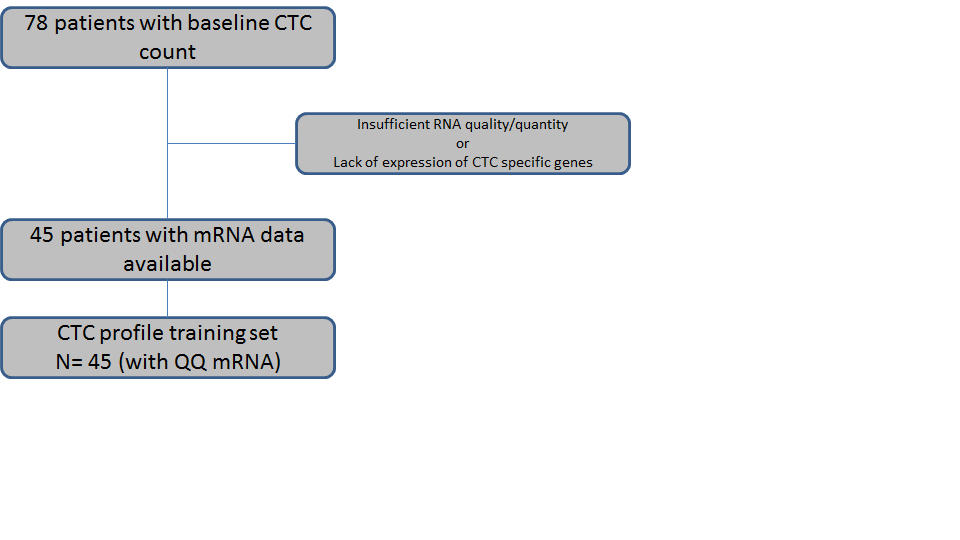

Supplement: Additional file 1: Figure S1. — Flowchart depicting the numbers of patients included and excluded from the study. (TIF 18.6 kb) [file 12885_2016_2155_MOESM1_ESM.tif]

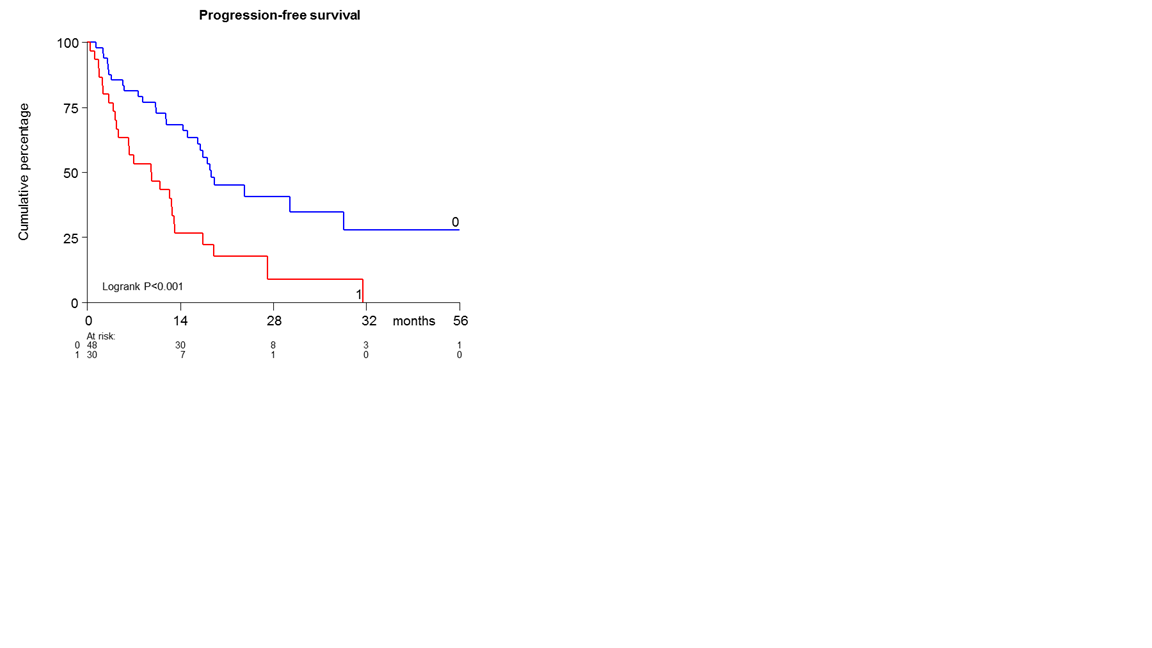

Supplement: Additional file 3: Table S1. — Cohort of in- (N = 45) and excluded (N = 33) patients and their clinico-pathological characteristics. (TIF 23 kb) [file 12885_2016_2155_MOESM3_ESM.tif]
